# Supplementary material for: Association of red blood cell distribution width-platelet ratio with mortality after coronary artery bypass grafting
Source: PeerJ. 2025 May 22;13:e19472. doi: 10.7717/peerj.19472 (PMC12103848; doi:10.7717/peerj.19472)
Supplement: Supplemental Information 2 — Abbreviations: BNP, B-type natriuretic peptide; LVEF, left ventricular ejection fraction; RDW, red blood cell distribution width; RPR, red blood cell distribution width-platelet ratio. [file peerj-13-19472-s002.docx]

Supplemental Information 1. Comparison of selected indicators between survivors and deaths in hospital.

| Variables | Deaths (n=45) | Survivors (n=1213) | χ^2^/Z | p value |
| --- | --- | --- | --- | --- |
| Platelet (K/μl) | 133 (83.5, 203) | 200 (157, 255) | -4.784 | < 0.001 |
| RDW (%) | 15.1 (13.65, 18.4) | 14.5 (13.6, 15.8) | -1.790 | 0.074 |
| RPR | 0.14 (0.07, 0.21) | 0.07 (0.06, 0.10) | -4.921 | < 0.001 |
| BNP (pg/ml) | 357 (205.5, 1079) | 260 (96.5, 710.5) | -2.251 | 0.024 |
| LVEF (%) | 42 (39, 48) | 46 (40, 52) | -2.181 | 0.029 |
| EuroSCORE | 5 (3, 7) | 4 (3, 5) | -1.908 | 0.056 |
| Previous stroke, n (%) | 11 (24.44) | 138 (11.38) | 7.096 | 0.008 |

Abbreviations: BNP, B-type natriuretic peptide; LVEF, left ventricular ejection fraction; RDW, red blood cell distribution width; RPR, red blood cell distribution width-platelet ratio.
